# Perinatal practice in extreme premature delivery: variation in Dutch physicians’ preferences despite guideline

**DOI:** 10.1007/s00431-016-2741-7

**Published:** 2016-06-01

**Authors:** Rosa Geurtzen, Jos Draaisma, Rosella Hermens, Hubertina Scheepers, Mallory Woiski, Arno van Heijst, Marije Hogeveen

**Affiliations:** Department of Pediatrics, Radboud university medical center Amalia Children’s Hospital, Nijmegen, The Netherlands; Scientific Institute for Quality of Care, Radboud university medical center, Nijmegen, The Netherlands; Department of Gynecology, Maastricht UMC+, Maastricht, The Netherlands; Department of Gynecology, Radboud university medical center, Nijmegen, The Netherlands

**Keywords:** Limits of viability, Prenatal counseling, Extreme prematurity, Treatment decisions, Resuscitation

## Abstract

Decisions at the limits of viability about initiating care are challenging. We aimed to investigate physicians’ preferences on treatment decisions, against the background of the 2010 Dutch guideline offering active care from 24^+0/7^ weeks of gestational age (GA). Obstetricians’ and neonatologists’ opinions were compared. An online survey was conducted amongst all perinatal professionals (*n* = 205) of the 10 Dutch level III perinatal care centers. Response rate was 60 % (*n* = 122). Comfort care was mostly recommended below 24^+0/7^ weeks and intensive care over 26^+0/7^ weeks. The professional views varied most at 24 and 25 weeks, with intensive care recommended but comfort care at parental request optional being the median. There was a wide range in perceived lowest limits of GA for interventions as a caesarian section and a neonatologist present at birth. Obstetricians and neonatologists disagreed on the lowest limit providing chest compressions and administering epinephrine for resuscitation. The main factors restricting active treatment were presence of congenital disorders, “small for gestational age” fetus, and incomplete course of corticosteroids.

*Conclusion*: There was a wide variety in individually preferred treatment decisions, especially when aspects were not covered in the Dutch guideline on perinatal practice in extreme prematurity. Furthermore, obstetricians and neonatologists did not always agree.

**Table:** 

**What is known:** • *Cross-cultural differences exists in the preferred treatment at the limits of viability* • *In the Netherlands since 2010, intensive care can be offered starting at 24* ^*+0/7*^ *weeks gestation*	
**What is new:** • *There was a wide variety in preferred treatment decisions at the limits of viability especially when aspects were not covered in the Dutch national guideline on perinatal practice in extreme prematurity*.

## Introduction

The advances in perinatal care have led to improved outcome in extreme prematurity. Gestational ages (GA) at which active treatment can be considered have decreased worldwide; however, a “gray zone” still remains [2, 6, 31, 35]. Therefore, in daily practice, several decisions have to be made about the initiation of care at 23–25 weeks GA. The key question is whether to initiate comfort care or active care. Factors that could be of influence are parental preferences and individual maternal or fetal characteristics. To support clinicians with decision-making in daily practice, several national guidelines on perinatal care are developed. Pignotti (2008), Gallagher (2014), and Guillen (2015) reviewed these guidelines and showed that 23 to 24 weeks of gestation are regarded as the gray zone of viability. In this gray zone, treatment decisions may be made using an individual approach and/or taking parental preferences into account. In some countries, this gray zone extends through to 25^+6⁄7^ weeks [10, 13, 28]. Not only guidelines differ, but also international, national, and local variations in actual practice do exist [4, 7, 12, 14, 17, 22, 23, 25, 30]. Furthermore, surveys amongst perinatal professionals revealed variation in (preferred) treatment decisions at the lower limits of viability, for example, decisions on performing a caesarian section (CS) and resuscitation [5, 9, 20, 24, 26, 32, 33].

Accompanying the decision whether or not to initiate care, several other choices have to be made, such as transfer to a specialized hospital, antenatal administration of corticosteroids, monitoring of the fetus, delivery mode, presence of the neonatologist at birth, and the extent of potential resuscitation. Guidelines do not always cover all these aspects [10, 13, 28]. In 2010, the national Dutch guideline on perinatal practice in extremely premature delivery lowered the limit offering intensive care from 25^+0/7^ to 24^+0/7^ weeks GA [8]. Unlike in other countries, in the Netherlands, this lower limit is rather strict (in contrast to, i.e., the American AAP guideline advocating for an individualized approach) and, in general, intensive care will not be offered below 24 weeks GA [4, 8, 10, 13, 28]. The Dutch guideline states “informed consent of parents is prerequisite in the decision whether or not to initiate care at 24 weeks GA”. It indicates that prognostic factors (such as weight, gender) can be taken into account in decision-making in individual cases. However, because the prognostic value of these factors is unknown for the Dutch population, no specific recommendations are given. Furthermore, the Dutch guideline recommends transfer to a tertiary center from 23^+4/7^ weeks GA for counseling, administration of corticosteroids from 23^+5/7^ weeks GA, and fetal monitoring with a CS can be considered in case of suspected fetal distress from 24^+0/7^ GA in which the specific risks and benefits for current and future pregnancies and delivery need to be discussed.

Like in most guidelines, variable operationalization is imaginable. Disagreement between perinatal professional (individuals and/or groups) on treatment decisions in extremely preterm gestations could potentially lead to a conflict in perinatal care [5, 9, 10, 32]. Therefore, our primary goal was to investigate Dutch physicians’ preferences on decisions about treatment options for an extremely premature neonate against the background of this guideline. Our secondary goal was to study potential differences between neonatologists and obstetricians.

## Materials and methods

### Study design

Cross-sectional, multicenter study (PreCo survey) using an online survey.

### Setting and study population

This study, the PreCo survey, is part of the larger PreCo study, which evaluates Dutch care on different levels in (imminent) extreme preterm birth, e.g., prenatal counseling and treatment decisions (clinicaltrials.gov, NCT02782650 & NCT02782637). This PreCo study is supported and followed by both the national associations of neonatology and obstetrics as well as the patient association.

The Dutch care for extreme premature births is centralized in 10 level III centers for perinatal care which all gave informed consent to participate in the current study. Surveys were sent to all fellows and senior staff members in both obstetrics and neonatology. Data were collected from July 2012 through October 2013, approximately 2 to 3 years after the introduction of the new guideline on perinatal practice in extreme premature delivery in the Netherlands.

### Survey design and data collection

The PreCo survey was developed in three stages. The first stage consisted of the development of a draft of the survey based on international literature about prenatal counseling; several prenatal counseling surveys that have kindly been shared with us [1, 3, 5, 18, 19, 27], observations from our previous study [11], and on public discussions generated by the Dutch guideline on perinatal practice in extreme premature delivery [8]. In the second stage, the survey was improved in two Delphi rounds; in the first round, the concept survey was extensively evaluated by four team members (two neonatologists, one obstetrician, and one pediatrician) and in the second round, independent perinatal experts (two neonatologists) pilot-tested the survey for clarity and content. In the third stage, the survey was adapted for both profession groups to exclude irrelevant questions and to optimize the participation rate. The PreCo survey required ~20 min to complete.

We were particularly interested in physicians’ preferences on treatment decisions. Therefore, we designed three questions: the first asking is for recommendations on whether or not to initiate intensive care at several extreme preterm GAs, the second asking is the personal lower limits in GA for various interventions potentially associated with extreme prematurity, and the third is on the importance of associated factors in recommending active treatment or not. We used a fictitious case of an “uncomplicated” extreme premature delivery to examine all these preferences. Finally, the last section of the entire survey contained demographical items such as age and years of experience.

An individual link to the online survey was sent to all participants. Three reminders were sent to non-responders. Survey results were anonymized before analysis. This study was waived by the local institutional review board.

**Table:** 

Characteristics of the fictitious case A consultation for prenatal counseling with an impending extreme premature delivery, singleton fetus, unremarkable history of pregnancy, average estimated fetal birth weight, unknown gender, no known congenital abnormalities, unremarkable social and medical history of parents, antenatal corticosteroids have been administered, and normal fetal heart rate registration.	

### Data analysis

Descriptive statistics were given as proportions of the respondents for that specific question. For comparison between obstetricians and neonatologists Chi-square (*Ӽ*^2^), Fisher exact, or Mann-Whitney U tests were used when applicable. Exact *p* values were provided, values <0.05 were considered significant. Statistical analyses were conducted using IBM SPSS Statistics (Version 20.0. Armonk, NY: IBM Corp).

## Results

We received 122 surveys from 205 eligible professionals; a response rate of 60 %. Each perinatal center was represented. Of those, 45 were from obstetricians and 77 from neonatologists. Of all 122 returned surveys, eight were only partially completed. Obstetricians had fewer years of experience than the neonatologists (Table 1). There was no influence of age or years of experience on the results, but some differences based on the institute of the participant did exist.

**Table 1:** Characteristics of professionals

	Obstetricians (*n* = 84 sent)	Neonatologists (*n* = 121 sent)
Response rate	54 %	64 %
Gender, % male	32 %	69 %
Having children (parent) % of those: parent of premature child (<27 weeks)	91 %	83 %
	0 %	2 %
Median age in years (q25–75*)*	40 (38–47)	45 (37–50)
Years of experience, median (q25–75)	5 (1–10)*	9 (4–17)

*p 0.02 (MWU)

**Fig. 1:**
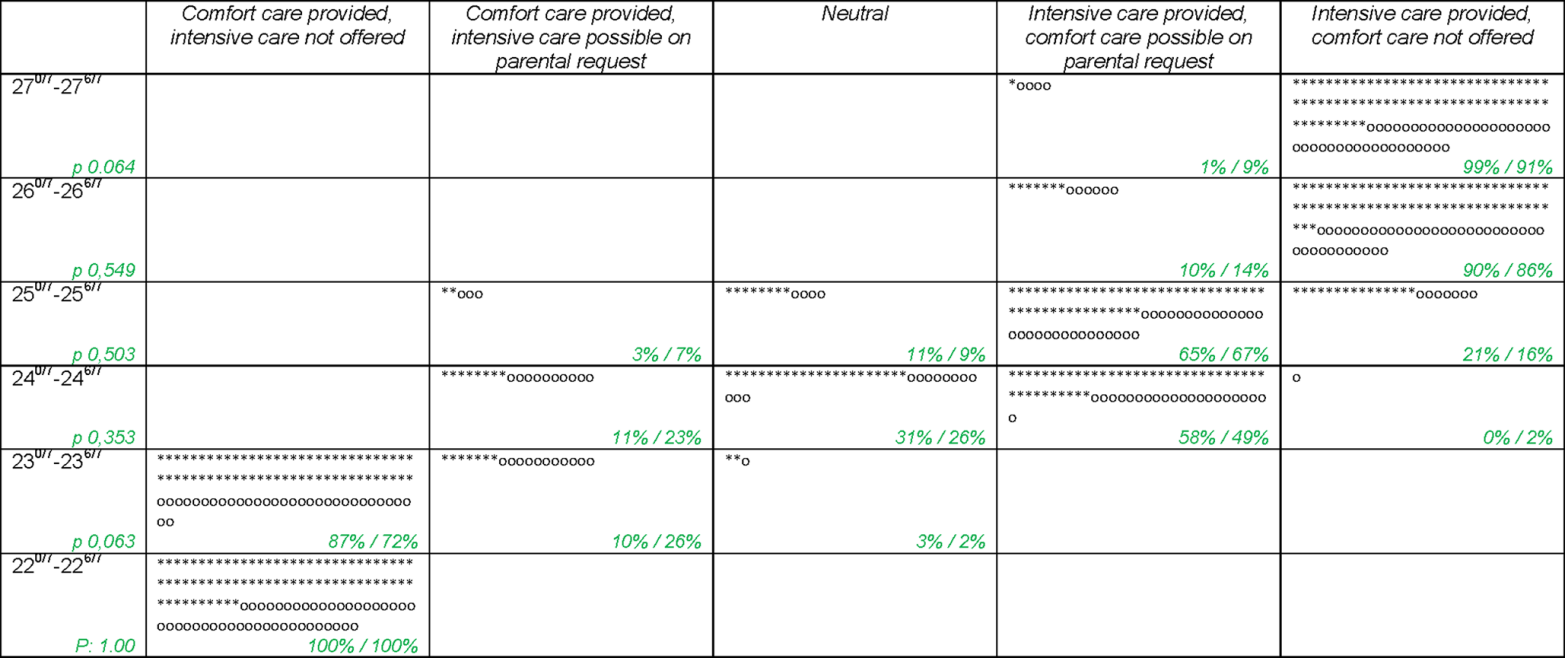
Recommendations for a pregnant woman who is about to deliver a fetus of the following gestational ages neonatologist = * obstetrician = o (individuals) xx %/xx % = proportion of neonatologist/proportion of obstetricians

Professionals gave their preferred recommendations about whether or not to initiate intensive care at each week of gestation, ranging from providing comfort care through providing intensive care (Fig. 1). At 22^+0–6/7^ weeks GA, recommending comfort care was the only option. For a birth at 23^+0–6/7^ weeks GA, 82 % recommended comfort care only, but some professionals (16 %) also agreed with intensive care at parental request. At 24^+0–6/7^ and 25^+0–6/7^ weeks GA, the majority (54 and 64 %, respectively) recommended intensive care with the ability of comfort care at parental request; however, there was variation in the given preferences. At 26^+0–6/7^ and 27^+0–6/7^ weeks GA, the vast majority (89 and 96 %, respectively) recommended intensive care without the possibility of comfort care—however, a minority would agree with comfort care at parental request (11 and 4 %, respectively). No significant differences were found for any of the GAs between obstetricians and neonatologists.

**Fig. 2:**
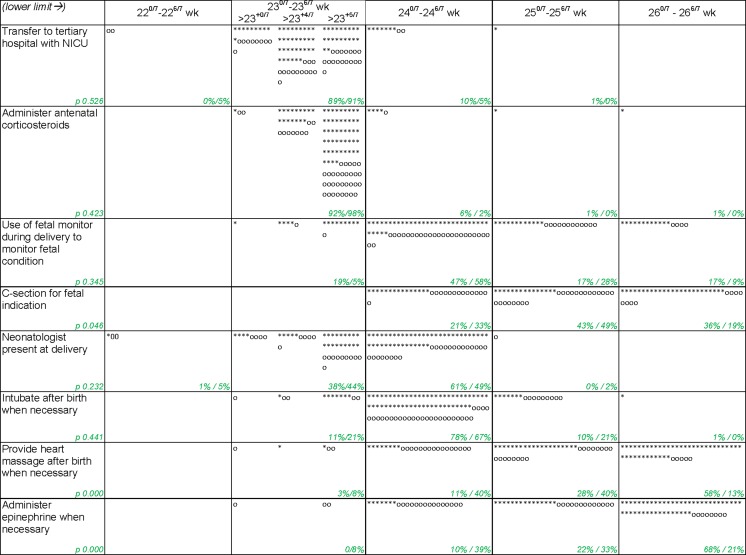
Personal limits of various interventions that could be taken around a possible premature delivery neonatologist = * obstetrician = o xx %/xx % = proportion of neonatologist/proportion of obstetricians

Professionals were asked for their personal lower limits for certain interventions or decisions at extreme prematurity (Fig. 2). Answer options ranged from “starting at 22^+0/7^ weeks GA” through “starting at 26^+0/7^ weeks GA”; only at 23 weeks more detailed answer options were available *(*23^+0/7^, 23^+4/7^, and 23^+5/7^ weeks GA*)*. There was variation between individuals up to 4 weeks. Medians in weeks GA (for this lower limit) were as mentioned below and interquartile ranges (IQR) are provided (obstetricians and neonatologists had the same median except where otherwise stated):

Transfer pregnant woman with imminent premature delivery to tertiary hospital with NICU: $$ \begin{array}{cc}\hfill {23}^{+4/7}\hfill & \hfill \left(IQR\; obstetricians\;{23}^{+0/7}\hbox{--} {23}^{+5/7} neonatologists\;{23}^{+4/7}\hbox{--} {23}^{+5/7}\right)\hfill \end{array} $$.

Antenatal administration of corticosteroids: $$ \begin{array}{cc}\hfill {23}^{+.5/7}\hfill & \hfill \left(IQR\; obstetricians\;{23}^{+4/7}\hbox{--} {23}^{+5/7} neonatologists\;{23}^{+5/7}\hbox{--} {23}^{+5/7}\right)\hfill \end{array} $$.

Use of fetal monitor during delivery for monitoring of the fetal condition: $$ \begin{array}{cc}\hfill {24}^{+0/7}\hfill & \hfill \left(IQR\; both\;{24}^{+0/7}\hbox{--} {25}^{+0/7}\right)\hfill \end{array} $$.

Perform a CS on fetal indication:$$ \begin{array}{cc}\hfill {25}^{+0/7}\hfill & \hfill \left(IQR\; obstetricians\;{24}^{+0/7}\hbox{--} {25}^{+0/7} neonatologists\;{25}^{+0/7}\hbox{--} {26}^{+0/7}\right)\hfill \end{array} $$.

Neonatologists have to be present at the delivery: $$ \begin{array}{cc}\hfill {24}^{+0/7}\hfill & \hfill \left(IQR\; both\;{23}^{+5/7}\hbox{--} {24}^{+0/7}\right)\hfill \end{array} $$.

Intubate after birth when necessary: $$ \begin{array}{cc}\hfill {24}^{+0/7}\hfill & \hfill \left(IQR\; both\;{24}^{+0/7}\hbox{--} {24}^{+0/7}\right)\hfill \end{array} $$.

Chest compressions after birth when necessary: obstetricians 25^+0/7^ and neonatologists $$ \begin{array}{cc}\hfill {26}^{+0/7}\left(p<0.01\right)\hfill & \hfill \left(IQR\; obstetricians\;{24}^{+0/7}\hbox{--} {25}^{+0/7} neonatologists\;{25}^{+0/7}\hbox{--} {26}^{+0/7}\right)\hfill \end{array} $$.

Administration of epinephrine after birth when necessary: obstetricians 25^+0/7^ and neonatologists $$ \begin{array}{cc}\hfill {26}^{+0/7}\left(p<0.01\right)\hfill & \hfill \left(IQR\; obstetricians\;{24}^{+0/7}\hbox{--} {25}^{+0/7} neonatologists\;{25}^{+0/7}\hbox{--} {26}^{+0/7}\right)\hfill \end{array} $$.

There was a disagreement between both professional groups with obstetricians having a significant lower threshold on providing chest compressions and administering epinephrine than neonatologist. Furthermore, significant differences between institutes were found on fetal monitoring, CS, chest compressions, and epinephrine, which may reflect local policies.

**Fig. 3:**
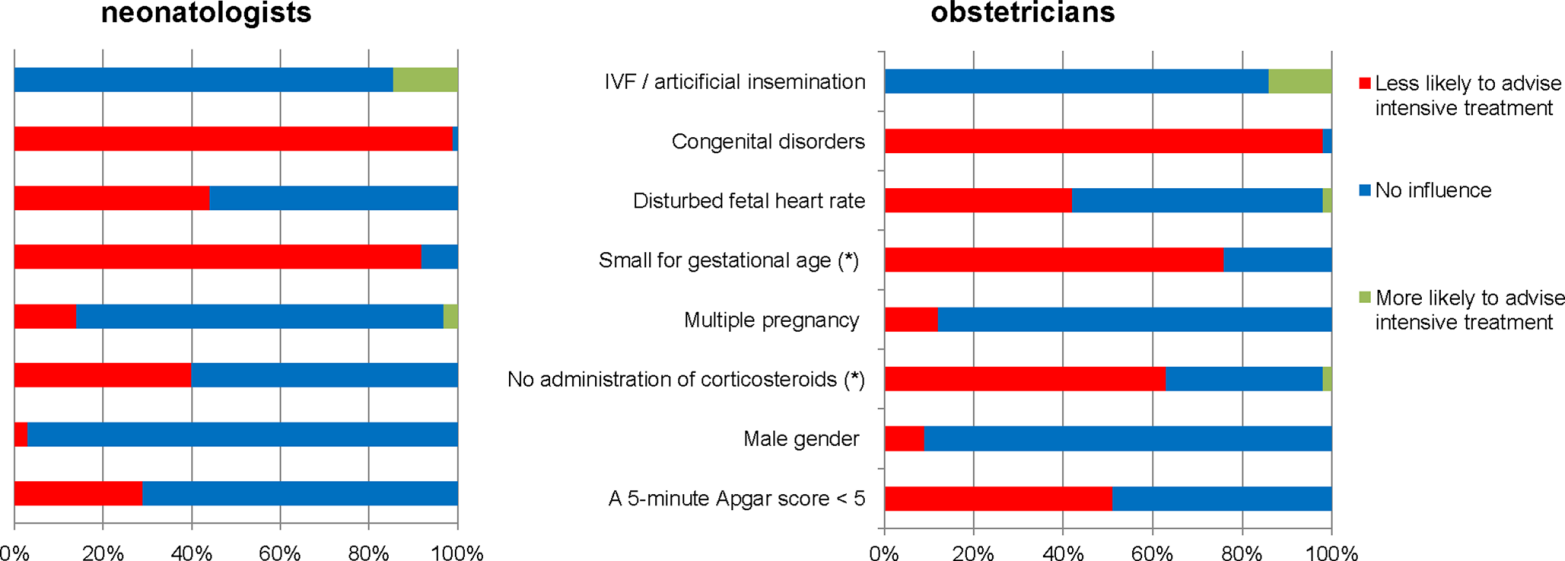
Factors influencing potential recommendation towards parents for initiating intensive treatment. (*)Significantly different between neonatologists and obstetricians: no corticosteroids p 0.012, SGA p 0.028

Figure 3 shows how certain factors could alter potential recommendations on initiating intensive care at 24^+0/7^ weeks GA. Ranked by the proportions of subjects being less likely to advise intensive treatment, congenital disorders was the strongest factor, followed by small for gestational age (SGA) infant, no antenatal administration of corticosteroids, and a disturbed fetal heart-rate. There were two items that differed between the two professional groups: first “no administration of corticosteroids”; this factor made 63 % of obstetricians versus 40 % of neonatologists less likely to advise intensive treatment (*p* = 0.033). Second for “SGA infant”, 92 % of neonatologists versus 76 % of obstetricians were less likely to advise intensive treatment (*p* = 0.028). Significant differences between institutes were found on male gender, multiple pregnancy, and disturbed fetal monitor, so again, some local preferences seem to exist.

## Discussion

This is the first study to assess professional preferences on treatment decisions in extreme prematurity in the Netherlands, particularly of neonatologists and obstetricians. There was a wide variation in preferred treatment decisions at the limits of viability, mostly when aspects were not covered in the Dutch national guideline on perinatal practice in extreme prematurity. This variation was shown between individual perinatal professionals on (a) the attitude towards the GA at which active treatment should be started, (b) the individual preferential lower limits of GA for certain interventions, and (c) the influence of additional patient characteristics on initiating care or not. Neonatologists’ and obstetricians’ opinions differed on the latter two.

### Recommendations on comfort versus intensive care

Regarding the large variation between perinatal professionals, our findings are comparable to Kaempf (2006) and Tomlinson (2010) who also discovered variety at various GA [18, 33]. At <24^+0/7^ weeks, comfort care is preferred to be recommended, consistent with the Dutch guideline. However, for some physicians, intensive care is an option at 23 weeks of gestation at parental request and, although being internationally practiced, it is not supported in the Dutch national guideline [8, 10, 28]. At 24 weeks, the Dutch guideline requests agreement of parents when initiating intensive care, and only one participant preferred recommending intensive care treatment only without accepting a potential parental request for comfort care. However, the majority did give the recommendation to provide intensive care with comfort care only as an option at parental request. The Dutch guideline describes that “intensive care can be offered” at 24 weeks GA, without explicitly giving an advice on whether to present this as default or present as neutral option next to comfort care. Our results show that at 24 weeks, variety in preferences exists. It is known that presenting delivery room options for extremely premature infants as default exerts a significant effect on decision makers [16]. At 25 weeks gestation, there is similar variety.

Notable is that some physicians approve comfort care on parental request at 26 and 27 weeks. The Dutch guideline does not explicitly cover this GA [8]. Also, internationally, 26 and 27 weeks of gestation are in general not being seen as the gray zone of viability anymore and the initiation of intensive care at these GA is being seen as standard of care [6, 10, 28, 31]. Kipnis argues that though treatment may offer a reasonable chance of a good outcome, there are situations in which neonatologists should nonetheless defer to parental nontreatment decisions’; however, no specific recommendation for 26 and 27 weeks GA is given [21]. Haward suggests that professional organizations should make guidelines on treatment decisions in extreme prematurity based on the best-interest principle [15]. To the best of our knowledge, there are no papers commenting on an upper limit in terms of GA where parental preferences can be followed including the legal aspects of this.

### Decisions or interventions associated with prematurity

The personal lower limit for certain treatment decisions associated with prematurity varied up to 4 weeks between individuals. It is known that personal preferences can influence counseling and decision-making [34]. For the interventions covered in the Dutch guideline, our results are fairly consistent with the guideline recommendations (transfer to a tertiary center at 23^+4/7^ weeks GA and administration of corticosteroids at 23^+5/7^ weeks GA). For these, 59 and 95 %, respectively, of all perinatal professionals have their lower limit at our below that mentioned GA, fulfilling the requirements of guideline. It should although be taken into account that the referring gynecologists (non-third line) were not questioned. Other interventions/decisions show large variation. According to the Dutch guideline, a CS at 24^+0/7^ weeks GA on fetal indication can be considered only in case of a spontaneously started delivery and after discussion with parents. Participants indicated 25^+0/7^ weeks of GA as median lower limit, with a wide variation. Tucker Edmonds recently showed that obstetricians had a personal cutoff for performing a CS at a later GA (median 25^+0/7^ weeks GA) than the institutional cutoff (median 24^+0/7^ weeks GA) [34]. Having a neonatologist present at delivery and intubation after birth when necessary both showed a median lower limit at 24^+0/7^ weeks of gestation, with relatively little variation; probably because these two items are seen as the minimum conditions that must be met when offering intensive care at 24^+0/7^ weeks (and implicitly covered in our guideline). However, there was a much wider range for providing chest compressions (when applicable) and administration of epinephrine. The reason for this wide range might be that they are not covered in the Dutch guideline. Compared to surveys from Finland, UK, and the USA, Dutch physicians seem to prefer almost all interventions at a later GA than their colleagues from mentioned countries [5, 32, 34].

Regarding the differences between obstetricians and neonatologists, for two items, obstetricians preferred significant lower limits than neonatologists (both chest compressions and epinephrine: median lower limit 25^+0/7^ weeks GA for obstetricians and 26^+0/7^ weeks GA for neonatologists). Apparently, obstetricians believe that intensive resuscitation can be provided at an earlier GA than their neonatal counterparts, which is contradictory to findings from England by Chan et al. (neonatal staff wished to be more interventional at earlier GA) and also from Finland by Taittonen et al. (pediatric personnel demonstrated more proactive attitudes to the treatment of a premature birth and baby than obstetric personnel) [5, 32]. Obstetricians do not normally perform these parts of resuscitation, so perhaps it was harder for them to comment on these items. However, obstetricians and neonatologists should participate as a team in taking care for mothers and newborns at the limits of viability; including agreement on the extent of potential neonatal resuscitation. Differences in opinions should be solved and no conflicts on care should arise [5, 10, 32].

### Role of associated factors

Individual prognostic factors do play a role in prenatal counseling at 24 weeks of GA for Dutch physicians, mainly a congenital disorder. Tyson et al. described four factors next to GA to have an impact on predicting outcome: birth weight, sex, (non)exposure to antenatal corticosteroids, and single/multiple gestation. These factors have a varying impact in this survey; for (male) gender and a multiple pregnancy <15 % of participants are less likely to advise intensive treatment. The two other factors from Tyson’s prediction model seem to have a greater impact; being SGA and no corticosteroids administered [35]. Although these two have an impact for both specialties, the proportion that agrees differs between them. We do not know why “no corticosteroids administered” is more important for obstetricians, and a “SGA infant” is more important for neonatologists. It is speculating, however, that these factors might be more visible within their specific expertise/field of work.

In contrast to Tyson, we did not try to quantify the prognostic factors and only asked for an influence. The Dutch guideline states that the factors from the Tyson model “can be taken into account”; however, since the value of these prognostic factors is unknown for the Dutch population, no specific recommendations are given. In 2014 though, 3 years after introduction of the guideline and after this survey was done, Dutch data showed that no antenatal corticosteroids, male gender, maternal age >35 years, Caucasian ethnicity, non-cephalic presentation, and birth outside a level III hospital were predictors for mortality (together with GA) in a prematurity cohort of GA 25^0^–31^6^ [29]. This survey shows that some factors were taken into account in prenatal consultation at 24 weeks GA. Nevertheless, we should allow for an individual approach to these prognostic factors, since the models are mostly not developed for counseling decisions and, most important, they do not predict an individual course. However, these factors might be helpful in addition to the GA to identify the potential range of outcome.

### Strengths and limitations

The strongest aspects of this study are the national level of the survey (all Dutch tertiary centers where included) representing our national situation. Also, the fact that most of the questions are directly related to content of the national guideline on perinatal practice makes it relevant for daily practice.

This study also has limitations. Some degree of selection-bias cannot be ruled out. The character of the survey (asking for recommendations to parents and personal lower limits for certain decisions) might not be representative for actual practice; however, no less relevant since it is known that, despite guidelines or local policies, personal preferences influence decision-making [34]. Because of the long inclusion period (16 months) effects of experience or learning cannot be ruled out, and it is unsure to what extent results from this Dutch cohort can be generalized to the international situation. However, many countries do have guidelines, so the general conclusion on variety between individuals and between professions despite guidelines might be applicable.

### Conclusions and future perspectives

This is the first study to asses physicians’ opinions on treatment decisions at the threshold of viability in the Netherlands. There was a wide variety in preferred treatment decisions at the limits of viability and in perceived lower limits of treatment between individual professionals. This variation was especially observed when aspects were not covered in the Dutch national guideline on perinatal practice in extreme prematurity. Furthermore, obstetricians and neonatologists disagreed on some aspects, particularly lower limits of GA for cardiac resuscitation and the influence of patient characteristics on initiating care. This variety and disagreement can lead to unwanted practice variation.

When items are covered in a guideline it seems to reduce, but not to exclude, variation. Especially when a guideline leaves room for interpretation, personal opinions will become more important. Revision of guidelines to cover more aspects might be a solution. However, more strict guidelines and recommendations that are based on national consensus need not interfere with an individualized approach, since making different choices based on patient characteristics and parental preferences are part of this consensus. The current study showed that in similar cases, dealing with different caregivers, different decisions can be made. At the limits of viability, it covers, by definition, decisions about life and death and practice variation is therefore even more unwanted.

## Abbreviations

CS: Caesarian section
GA: Gestational age(s)
IQR: Interquartile range
SGA: Small for gestational age
